# Social anxiety but not callous-unemotional traits predicts shame coping in conduct disorder

**DOI:** 10.3389/fpsyt.2026.1813691

**Published:** 2026-06-11

**Authors:** Laura M. Derks, Kelly Melvin-Hilmer, Martin Holtmann, Tanja Legenbauer

**Affiliations:** Research Department, LWL University Hospital Hamm for Child and Adolescent Psychiatry, Psychotherapy and Psychosomatic, Ruhr-University Bochum, Hamm, Germany

**Keywords:** callous-unemotional traits, conduct disorder, shame coping, shame proneness, social anxiety

## Abstract

**Introduction:**

Conduct disorders are characterized by emotional dysregulation. Both callous-unemotional traits and social anxiety are heightened in conduct disorder patients and are associated with different mechanisms of emotion regulation. Previous evidence has proposed that secondary emotions, such as shame, might also be affected in conduct disorder and that callous-unemotional traits and social anxiety might be related to shame as well as to shame coping. Therefore, the current study investigated links between callous-unemotional traits, social anxiety, shame proneness, and shame coping in adolescent inpatients with conduct disorder.

**Methods:**

Forty adolescent inpatients with conduct disorders (*M* = 12.4, *SD* = 1.4) filled in questionnaires on callous-unemotional traits, social anxiety, shame proneness, and shame coping. Correlational and regression analyses, as well as mediation analyses were performed.

**Results:**

Callous-unemotional traits were not associated with any other construct. Social anxiety showed positive correlations with shame proneness and internalizing as well as externalizing shame coping. Social anxiety was also a significant predictor of internalizing shame coping while controlling for shame proneness and callous-unemotional traits. No predictors emerged for externalizing shame coping. Mediation analyses confirmed that neither shame proneness nor social anxiety mediated the relationship between CU traits and shame coping, as CU traits were not significantly associated with either variable.

**Discussion:**

The findings suggest that social anxiety plays a key role in internalizing shame coping in conduct disorder patients. CU traits appear to be unrelated to shame proneness and shame coping, either directly or indirectly, in conduct disorder.

## Introduction

The last two decades have seen a proliferation of research findings indicating that “callous-unemotional traits” (CU traits) – defined as traits characterized by unemotionality, callousness, and uncaringness (1) – exert a significant influence on the behavior and emotions of children and adolescents (2, 3). These traits appear to be particularly prominent in children and adolescents diagnosed with conduct disorder (CD) and to be more likely to persist into adulthood in these individuals (4). The presence of CU traits has been shown to exert a detrimental effect on the emotional well-being, social interaction, and general mental health of children diagnosed with CD, with the result that therapeutic outcomes are often less than optimal (5).

Therefore, research has increasingly focused on the impact of CU traits on emotional reactivity and regulation in children and adolescents with CD and CU traits (6–9). These studies have reported, for instance, difficulties in regulating negative emotions such as anger or sadness, and an association between such emotion regulation problems and a higher intensity of aversive or negative responses. Interestingly, these responses appear to be affected by levels of trait anxiety – while individuals with CU traits and low trait anxiety show difficulties in responding appropriately to the distress of others, those with CU traits and high trait anxiety show stronger arousal reactivity to emotional stimuli as well as higher impulsivity and negative affect (5, 10, 11). Furthermore, interpretation biases in ambiguous situations have been found in adolescents with CU traits (12, 13).

Hence, a substantial body of research has explored the relationship between anxiety and CU traits in children and adolescents. Given that CU traits exert the most substantial effects on social behavior, they might show an especially prominent relationship with social anxiety (14–16). Evidence, however, is mixed and some authors have suggested that both individuals with high (CU+) and low (CU-) CU traits can exhibit heightened social anxiety (17, 18).

Research has shown that differences between children with and without CU traits emerge early in development (19, 20). In particular, such temperament-based differences are closely tied to the development of conscience, primarily shaped by guilt and empathy (21). Findings of a negative correlation between CU traits and prosocial behavior (22, 23) suggest a broader link between CU traits, moral development, and emotional responses like shame and social anxiety (3). Two previous studies have addressed perceived shame — a “moral emotion” — and CU traits, with one study suggesting no correlation in prepubertal children (24) and the other reporting a significant correlation between shame and CU traits in older adolescents (25). Interestingly, shame has also been found to be significantly related to social anxiety (26, 27). Hence, addressing moral emotions in this field can add to the existing literature by further exploring the underlying mechanisms of the possible negative link between CU traits and (social) anxiety.

‘Moral emotions’ are defined as those that are oriented towards societal norms and cultural conditions (28). They arise when an individual evaluates or judges their own actions or behavior in the light of societal values, potentially resulting in feelings of guilt or shame if the individual perceives themself not to have met societal expectations. Shame is frequently characterized in the extant literature as being “painful and intense”, to the extent that it necessitates specific coping strategies (29). Specifically, two internalizing and two externalizing types of shame coping mechanisms have been identified, with internalizing types including withdrawal from emotionally stressful situations and self-directed aggressive behavior, and externalizing types including a tendency to attack others and to avoid triggering situations in the future (30). A study in older adolescents (age 16–21 years) found that these shame coping mechanisms were related to CU traits, and a positive correlation was identified between the level of CU traits and the externalizing poles of the shame coping mechanism (31). However, to date, the research shows no consensus on how shame coping influences coping behavior in children and adolescents with CU traits, and no study has addressed the association between shame coping, social anxiety, and CU traits. Research in adults has reported positive relationships with externalizing coping strategies in individuals with high CU traits, and in part, an inverse relationship between CU traits and internalizing coping strategies, with individuals with higher CU traits being less likely to endorse internalizing mechanisms (32). However, the above-mentioned study by Nyström and Mikkelsen (31) found no difference in the use of internalizing strategies according to the level of CU traits but did confirm a positive association between CU traits and externalizing strategies in older adolescents. In summary, the results regarding a potential link between CU traits and shame coping are largely inconclusive, and research examining this link, particularly among children and adolescents with CD and low CU traits, is sparse.

The present study sought to ascertain the extent to which patients with CD differ in terms of the moral emotion of shame and the associated coping strategies depending on the individual manifestation of CU traits. Additionally, we examined the triangular relationship between CU traits, emotional shame coping, and social anxiety. Based on previous preliminary findings, we assumed that higher CU traits would be associated with less shame proneness, lower social anxiety, lower internalizing shame coping, and higher externalizing shame coping. We further hypothesized that the relation between CU traits and shame coping would be mediated by the level of social anxiety and by shame proneness.

## Materials and methods

### Participants

Participants were recruited as part of a larger study examining children and adolescents with CD (https://doi.org/10.1186/s13034-024-00831-y). The data used for this study consisted of baseline data that were collected before the start of the experiment conducted in the larger study. The current sub-study analyzed a subsample of forty male inpatients who were recruited at the LWL University Hospital for Child and Adolescent Psychiatry Hamm, Germany and had been diagnosed with CD based on the International Classification of Diseases [10^th^ ed.; F90.1, F91.-, F92.- (33);]. Participants were aged between 10 and 14 years (*M* = 12.4, *SD* = 1.4). Besides a diagnosis of CD, as confirmed by a diagnostic interview with participants’ caregivers, the inclusion criteria were an intelligence quotient (IQ) ≥ 85 and sufficient German-language proficiency to understand the questionnaires. Exclusion criteria were physical limitations in terms of walking and eyesight, as well as epilepsy, which would have impeded participation in the virtual reality task of the aforementioned larger study. Additionally, psychiatric comorbidities that might have impeded study participation (i.e., posttraumatic stress disorder (PTSD), pervasive developmental disorders, psychotic symptoms, and acute suicidality) led to exclusion. **Table 1** presents the descriptive statistics of the sample in more detail.

**Table 1:** Descriptive characteristics of the sample.

Variable	*M*	*SD*
Age	12.44	1.37
IQ	96.5	7.04
No. of comorbidities	1.85	0.08
Variable	*n*	*%*
Main diagnosis		
Hyperkinetic CD (F90.1)	2	5.0
CD (F91.-)	2	5.0
Combined disorder of conduct and emotion (F92.-)	34	85.0
Other	2	5.0
Secondary diagnoses		
Disturbance of activity and attention (F90.0)	13	32.5
Hyperkinetic CD (F90.1)	4	10.0
Comorbidities		
Yes	25	62.5
No	15	37.5
Medication		
None	22	55.0
Stimulants	10	25.0
Other	2	5.0
Combination	6	15.0

IQ, intelligence quotient as measured with the short version of the Wechsler Nonverbal Scale of Abilities [WNV (46);]; CD, conduct disorder.

^a^ Diagnoses were retrieved from the participants’ digital hospital records and based on the ICD-10.

^b^ Only diagnoses with a prevalence of ≥ 10% in the current sample are reported in the table.

### Measures

### Demographic variables

Demographic variables of interest (i.e., age, (number of) main and secondary diagnoses, medication, and results of prior intelligence tests) were retrieved from the participants’ digital hospital records.

### CU traits

The German self-report version of the Inventory of Callous-Unemotional Traits[(ICU; original version by (34, 35); German version by (35)] was used to measure CU traits. The questionnaire consists of 24 items rated on a 4-point Likert scale ranging from 0 (“not at all true”) to 3 (“definitely true”). The items cover the three CU facets of unemotionality (i.e., reduced emotional expression), uncaringness (i.e., indifference towards own performance and feelings of others), and callousness (i.e., lack of empathy, remorse, and guilt). A total score can be calculated by summing all items. The German version of the ICU has been validated for children in the included age range (36). In the current study, the reliability of the total score was acceptable, with Cronbach’s α = .71.

### Shame coping

Shame coping style was measured using the German version of the self-report Compass of Shame Scale [CoSS-d, 5^th^ version; original version by (30); German version by (37)]). The questionnaire describes 12 potentially shame-inducing scenarios with four possible reactions that are each linked to one pole of the CoSS (i.e., “withdrawal”, “attack self”, “avoidance”, “attack others”), resulting in a total of 48 items. Each item captures the frequency with which participants would react to the described scenario in the depicted way on a 5-point Likert scale ranging from 0 (“never”) to 4 (“almost always”). In a validation study by (38), the two subscales of internalizing (i.e., “withdrawal” and “attack self”) and externalizing shame coping (i.e., “avoidance” and “attack others”) were formed out of the four poles. For the COSS, the self-administered versions are validated for children ≥ 12 years in Portuguese (39) and ≥ 14 years in German (37). The reliability in the current study was excellent for the internalizing subscale, with Cronbach’s α = .92, and good for the externalizing subscale, with Cronbach’s α = .80.

### Shame proneness

Shame proneness was measured using the German version of the self-report Test of Self-Conscious Affect for Adolescents [TOSCA-A; original version by (40); German version by (41)]). The questionnaire describes 15 daily-life scenarios along with four or five possible reactions, which are believed to capture the individual tendency to experience shame, guilt, or indifference, or to externalize one’s feelings. Respondents are asked to indicate their likelihood of reacting in the manner indicated on a 5-point Likert scale ranging from 1 (“very unlike me”) to 5 (“very like me”). Only the shame subscale was employed in the present study. The German version of the TOSCA has been validated for children in the included age range (42). The reliability for this subscale in the present sample was good, with Cronbach’s α = .81.

### Social anxiety

Social anxiety was measured using the German version of the self-report Social Anxiety Scale for Children [SASC-R-D; original version by (43); German version by (44)]. The SASC-R-D contains 22 items rated on a 5-point Likert scale ranging from 1 (“never”) to 5 (“always”). The items describe fear of negative evaluation, social avoidance, and distress in various social situations. A total score can be derived by summing 18 of the 22 items. The German version of the SASC-R-D has been validated for children in the included age range (45). In the current study, the reliability of the total score was excellent, with Cronbach’s α = .91.

### Control variables

Intelligence screening was carried out using the short version of the Wechsler Nonverbal Scale of Abilities [WNV (46);]. The WNV is a nonverbal measure suitable for children and adolescents aged 8 to 21 years. The short version contains the two subscales “Matrices” and “Spatial Span”, from which a total score is derived.

The diagnosis of CD was confirmed using the Diagnostic Interview for Mental Disorders in Children and Adolescents [Kinder-DIPS (47);], a structured clinical interview assessing common mental disorders based on ICD-10 criteria. In the present study, we only employed the CD subsection, which was conducted with the participant’s primary caregiver.

### Procedure

All study materials (i.e., information, informed consent, questionnaires, instructions) were presented in German. Psychiatric inpatients with a CD diagnosis were asked if they wished to participate in the study. If they expressed an interest in study participation, their legal guardians were contacted and informed about the study. Both patients and their legal guardians were informed about the study verbally and in writing and had the opportunity to ask any questions. After informed consent was provided by both parties, the diagnostic interview was conducted with the primary caregiver. If the CD diagnosis was confirmed, participants then completed IQ screening using the WNV, if no prior IQ measurement had occurred within the last year. Subsequently, participants completed the study questionnaires (ICU, SASC, TOSCA, CoSS).

### Data analysis

Data preparation and analysis were conducted using IBM SPSS Statistics 30.0.0.0. Missing values on questionnaires were analyzed and found to be missing completely at random (Little’s MCAR tests were conducted for each questionnaire separately; all *p*s >.05). Missing data were replaced with the individual mean value on the respective questionnaire’s subscale. Descriptive statistics were calculated for the variables of interest. Normality tests (Shapiro-Wilk and Kolmogorov-Smirnov tests) were conducted for the ICU and SASC total scores, the TOSCA shame subscale, and the CoSS internalizing and externalizing subscales, and revealed no significant deviation from normality (all *p*s >.05). First, associations of IQ and medication status with CU traits, social anxiety, shame proneness, and shame coping were assessed to evaluate their potential as confounding variables. Second, two-tailed Pearson correlation analyses were performed, followed by the calculation of two 3-step hierarchical regressions to assess the triangular relationship between CU traits, social anxiety, and shame coping. Shame proneness was entered in step 1, as it represents the construct most proximal to the outcome (i.e., shame coping). Given the lack of clear empirical guidance regarding the relative ordering of CU traits and social anxiety, these variables were entered in steps 2 and 3, respectively, to examine their incremental contributions beyond shame proneness. To account for potential effects of predictor ordering, the analyses were repeated with reversed entry (i.e., social anxiety entered in step 2 and CU traits in step 3). Internalizing and externalizing shame coping were examined as separate dependent variables. Subsequently, the mediating effects of shame proneness and social anxiety in the relationship between CU traits and shame coping were assessed in four mediation analyses with Hayes’ PROCESS v4.3 macro.

An *a priori* power analysis was conducted using G*Power for the hierarchical multiple regression analyses. Based on this analysis, a sample size of *N* = 40 was considered sufficient to detect medium to large effect sizes.

The achieved statistical power was 1-*ß* > 0.86 to detect large predictor effects (*f2* = 0.35), 1-*ß* = 0.47 for medium-sized effects (*f2* = 0.15), and 1-*ß* = 0.10 for small effects (*f2* = 0.02).

## Results

Descriptive statistics were calculated for the questionnaire data on CU traits, social anxiety, shame proneness, and shame coping, and are presented in **Table 2**. The mean score for CU traits lay above the recommended cut-off [cf (48)], while the mean score for social anxiety lay below the recommended clinical cut-off [cf (43)]. Since there are no recommended cut-offs for shame proneness and shame coping, the scores were compared to those reported in validation studies. Shame proneness in the current sample was low compared to school samples within the same age range [cf (49)]. Scores for both internalizing and externalizing shame coping were higher than those reported in the original validation study (50).

**Table 2:** Descriptive statistics of CU traits, social anxiety, shame proneness, and shame coping.

Variable	*M*	*SD*	Min	Max
CU traits^a^	31.08	7.85	17	55
Social anxiety	40.10	13.22	21	74
Shame proneness	35.50	9.55	18	62
Shame coping				
Internalizing	56.38	18.47	24	100
Externalizing	57.28	12.54	34	87

CU traits were measured using the ICU, social anxiety using the SASC-R-D, shame proneness using the TOSCA-A, and internalizing and externalizing shame coping using the CoSS-d (5^th^ edition).

^a^Mean score on the questionnaire is above the clinical cut-off for this measure.

### Control analyses

Associations of IQ and medication status with CU traits, social anxiety, shame proneness, and shame coping were assessed using two-tailed correlation analyses and univariate ANOVAs to evaluate their potential as confounding variables. As all *p*s were >.05, IQ and medication status were not included as covariates in the subsequent analyses.

### Correlational analyses

Two-tailed correlation analyses were performed between CU traits, social anxiety, shame proneness, and shame coping (see **Table 3**). Whereas CU traits did not correlate with any of the other measures, social anxiety showed strong positive correlations with shame proneness and internalizing shame coping and a moderate positive correlation with externalizing shame coping. The two types of shame coping also showed a strong positive correlation with each other.

**Table 3:** Correlation analysis between study variables.

Variable	CU traits	Social anxiety	Shame proneness	Internalizing coping	Externalizing coping
CU traits	–	.019	-.007	.101	.184
Social anxiety	.019	–	.746^***^	.824^***^	.478^**^
Shame proneness	-.007	.746^***^	–	.728^***^	.503^***^
Internalizing coping	.101	.824^***^	.728^***^	–	.623^***^
Externalizing coping	.184	.478^**^	.503^***^	.623^***^	–

CU traits were measured using the ICU, social anxiety using the SASC-R-D, shame proneness using the TOSCA-A, and internalizing and externalizing shame coping using the CoSS-d (5^th^ edition).

^***^p <.001, ^**^p <.01.

### Hierarchical regressions

Two hierarchical multiple linear regression analyses (internalizing vs. externalizing shame coping; **Table 4**) were performed with shame proneness entered as a predictor in step 1, CU traits entered as a predictor in step 2, and social anxiety entered as a predictor in step 3 to gain a better understanding of the associations between CU traits, social anxiety, and shame coping while controlling for shame proneness.

**Table 4:** Hierarchical multiple regression analyses predicting shame coping from shame proneness, CU traits, and social anxiety.

Variable	*B*	BCa 95% CI for *B*		*SE B*	*β*	*R²*	*Δ R²*
		LL	UL				
Internalizing shame coping								
Step 1						.529^***^	
Constant	6.449	-6.656	19.029	7.342			
SP	1.406^***^	0.993	1.880	0.210	.728		
Step 2						.541^***^	.011
Constant	-1.345	-33.491	17.656	13.951			
SP	1.408^***^	0.905	2.005	0.250	.728		
CU	0.249	-0.430	1.081	0.359	.106		
Step 3						.716^***^	.175^***^
Constant	-3.299	-29.686	14.212	11.138			
SP	0.501	-0.217	1.181	0.297	.259		
CU	0.214	-0.402	0.846	0.289	.091		
SA	0.878^***^	0.513	1.216	0.196	.629		
Externalizing shame coping							
Step 1						.253^***^	
Constant	33.856^***^	21.684	45.990	6.943			
SP	0.660^**^	0.256	1.106	0.201	.503		
Step 2						.288^**^	.035
Constant	24.512^*^	2.747	39.481	11.250			
SP	0.661^**^	0.213	10.193	0.228	.504		
CU	0.299	-0.212	0.926	0.243	.187		
Step3						.310^**^	.022
Constant	24.041^†^	1.702	39.644	11.626			
SP	0.443^†^	-0.089	1.006	0.251	.337		
CU	0.290	-0.208	0.905	0.233	.182		
SA	0.212	-0.211	0.630	0.210	.223		

CI, confidence interval; *LL*, lower limit; *UL*, upper limit; SP, shame proneness subscale of the TOSCA-A; CU, Inventory of Callous-Unemotional Traits (ICU) total score; SA, Social Anxiety Scale for Children (SASC-R-D) total score. The 95% bias-corrected and accelerated confidence intervals and standard errors were based on 1000 bootstrap samples.

^***^p <.001 ^**^p <.01 ^*^p <.05 ^†^*p* <.10.

All three steps of the *regression model for internalizing coping* were significant (model 1: *F*(1, 39) = 42.752, *p* = <.001, *R²* = .529, model 2: *F*(2, 39) = 21.775, *p* = <.001, *R²* = .541, model 3: *F*(3, 39) = 30.261, *p* = <.001, *R²* = .716). While shame proneness was a significant predictor in the first two models, social anxiety was the only significant predictor of internalizing shame coping in the final model. Specifically, a 1-point increase in social anxiety scores led to an increase of 0.88 in internalizing shame coping scores.

All three steps of the *regression model for externalizing coping* were significant (model 1: *F*(1, 39) = 12.841, *p* = <.001, *R²* = .253, model 2: *F*(2, 39) = 7.468, *p* = .002, *R²* = .288, model 3: *F*(3, 39) = 5.383, *p* = .004, *R²* = .310). Shame proneness was a significant predictor in the first two models, whereas the final model did not reveal any significant predictors of externalizing shame coping.

Both regression analyses were repeated with social anxiety entered in step 2 and CU traits in step 3, which did not meaningfully change the results. Again, all three steps of the *regression model for internalizing coping* were significant. While shame proneness was a significant predictor in the first two models, social anxiety was the only significant predictor of internalizing shame coping in the final model. Also for *externalizing coping*, all three steps of the regression model were significant, with shame proneness being a significant predictor in the first model. No significant predictors emerged in the second or the final model.

### Follow-up analyses: mediating effects of shame proneness and social anxiety

To test for potential mediation effects of shame proneness and social anxiety, four mediation analyses were conducted with PROCESS.

First, we tested the mediating effect of *shame proneness* on the relationship between CU traits and both externalizing and internalizing shame coping. The first step of our analyses revealed a non-significant effect of CU traits on shame proneness, *b* = -0.0081, *p* = .967. The second step revealed a significant effect of shame proneness on internalizing shame coping, *b* = 1.4077, *p* <.001, and on externalizing shame coping, *b* = 0.6613, *p* = .009. The third step showed that the direct effect of CU traits on shame coping was non-significant, with *b =* 0.2492, *p* = .348 for internalizing shame coping, and *b* = 0.2988, *p* = .189 for externalizing shame coping. Thus, our data contradict a possible mediation of the relationship between CU and shame coping through shame proneness.

Second, we tested the mediating effect of *social anxiety* on the relationship between CU and both internalizing and externalizing shame coping. The first step of our analyses revealed a non-significant effect of CU traits on social anxiety, *b* = 0.0317, *p* = .908. The second step revealed a significant effect of social anxiety on internalizing shame coping, *b* = 1.1487, *p* <.001, and on externalizing shame coping, *b* = 0.4505, *p* = .002. The third step revealed that the direct effect of CU traits on shame coping was non-significant, with *b =* 0.2014, *p* = .359 for internalizing shame coping, and *b* = 0.2791, *p* = .225 for externalizing shame coping. Thus, our data contradict a possible mediation of the relationship between CU and shame coping through social anxiety.

## Discussion

The present study is the first to investigate the relationship between CU traits, social anxiety, shame proneness, and shame coping. Unlike social anxiety, which showed a strong correlation with shame proneness and internalizing shame coping and a moderate correlation with externalizing shame coping, CU traits were not associated with shame, its coping mechanisms, or social anxiety. Social anxiety was the only significant predictor of internalizing shame coping, while no predictors emerged for externalizing coping. Mediation analyses confirmed that neither shame proneness nor social anxiety mediated the relationship between CU traits and shame coping, as CU traits were not significantly associated with either variable. These findings highlight the association between social anxiety and internalizing shame coping, while CU traits appear to be unrelated to shame coping, either directly or indirectly.

First of all, regarding the overall lack of relationship between CU traits and shame, one possible explanation might be that moral emotions like shame and guilt develop later than basic emotions, and require self-awareness, sensitivity to others’ judgments, and an understanding of social norms, which typically emerge between the ages of three and eight years (28). CDs can be regarded as emotional developmental disorders and are often associated with dysfunctional parenting and antisocial peer influences (51). These factors may hinder the development of moral emotions in children with social behavior disorders, resulting in the observed non-significant correlation between shame and CU traits in childhood. Other researchers have likewise reported this lack of association: A US study in prepubertal children by Lotze et al.24 (24) also found no correlations between shame proneness, which was also assessed by the TOSCA-A, and the level of CU traits. In contrast, a Portuguese study reported a significant correlation between shame and CU traits in older adolescents attending secondary school (25). The higher average age of these participants may serve as one explanation for the differing results, suggesting a link between CU traits, impaired emotional development, and shame in adolescents but not in younger children, potentially due to delayed emotional maturation.

Second, it is important to address the non-significant associations between social anxiety and CU traits: While some previous studies indicated a negative relationship between CU traits and social anxiety (15, 23), others reported a positive correlation (52, 53). Supporting the present findings, there is also evidence showing no association between CU traits and social anxiety (35, 54, 55). Again, the discrepant findings may be due to sample characteristics: Dolan and Rennie (56) examined incarcerated adolescents with CD and found that higher CU traits, measured using the Psychopathy Checklist – Youth Version (PCL-YV), correlated with lower social anxiety. Similarly, Frick et al.57 (57) found a strong negative correlation between CU traits and social anxiety, but only after controlling for CD. Thus, besides age, the level of CD may also interact with and impact the relation between social anxiety and CU traits. Indeed, studies that controlled for the level of CD problems found the expected negative relation between CU traits and social anxiety (23, 57–60).

Third, our finding of a lack of association between CU traits and shame coping styles does not correspond to previous research. For example, a Swedish study found a positive correlation between externalizing shame coping and CU traits in children and adolescents from the general population (31). Similarly, a Portuguese study reported increased use of strategies from the “attacking others”-subscale of the CoSS in male adolescents from foster care or juvenile detention facilities, while other coping strategies were less common (39). However, the latter study did not assess psychopathological data, rendering it unclear whether participants had CD and thus limiting the comparability of the findings with the present study. Nevertheless, previous research suggests that individuals show heterogeneous shame coping styles, often involving both internalizing and externalizing strategies (38). Additionally, the diagnosis of CD, linked to poor emotion regulation (61), may contribute to overlapping coping behaviors. The strict categorization of shame coping into internalizing or externalizing strategies might not accurately reflect coping mechanisms in individuals with CD. Furthermore, emotion processing deficits in CD, such as difficulties in recognizing others’ emotions (62, 63) and a lack of empathy, a key CU trait, might affect the interpretation of CoSS scenarios and consequently lead to biased questionnaire responses.

Next to the aforementioned factors, the lack of associations between CU traits and the other measures might also stem from measurement or sampling problems. Due to the clinical nature of our sample, characterized by high levels of symptoms that require inpatient treatment and significant comorbidity, limited variance in CU traits might have attenuated the associations. Moreover, although the use of the ICU has been validated in children and young adolescents, there are no specific validation studies in youth with ADHD. This comorbidity might lead to psychometric challenges, including potential limitations in the sensitivity of the questionnaires.

Finally, the associations of social anxiety with shame, as well as shame coping, need to be addressed. In the present study, we found a significant positive correlation between shame proneness and social anxiety, which corresponds to previous research, including a meta-analysis (27). Given that social anxiety involves heightened self-criticism and fear of negative evaluation, an increased correlation with shame can be expected. The present findings additionally revealed a strong positive correlation between social anxiety and both internalizing and externalizing shame coping strategies, aligning with a previous study that reported a positive link between internalized shame and avoidance — one of the internalizing coping strategies of the CoSS — which was mediated by social anxiety (64). With regard to externalizing coping, a Dutch study found that students displaying more externalizing coping also reported higher social anxiety levels than did those displaying internalizing strategies (65).

It is important to note that the present study examined a clinical sample of children and adolescents receiving inpatient treatment for CD, characterized by pronounced externalizing behavior. As discussed, CD is associated with significant difficulties in emotion regulation. Given that shame and guilt are complex self-conscious emotions (28), affected individuals may rely on maladaptive coping strategies to manage them.

From a clinical perspective, the findings underscore the heterogeneity of CD. Although CD is primarily conceptualized as an externalizing disorder, the present results highlight the relevance of internalizing processes in this population. In particular, the robust association between social anxiety and internalizing shame coping suggests that clinicians should routinely assess social anxiety, as well as shame proneness and coping styles, as part of comprehensive diagnostics. Moreover, the findings point to the potential value of targeting maladaptive coping with internalizing emotions in treatment. Interventions addressing social-evaluative fears and promoting more adaptive emotion regulation strategies may be beneficial, particularly in inpatient settings where structured therapeutic support is available.

### Limitations

Several limitations of this study should be considered when interpreting the findings. First, the sample included both prepubescent and pubescent participants (ages 10–14), which, combined with developmental differences in guilt and shame processing (66) and in emotion regulation deficits in CD, may have rendered it difficult for participants to distinguish between these emotions. Moreover, CD and CU traits are associated with higher levels of manipulative traits (67). This may affect their responses on self-administered questionnaires, highlighting the importance of replicating the observed associations using additional measures and multiple informant sources. Second, data collection was part of a larger study, which required participants to complete an extensive and linguistically demanding questionnaire battery. The high rate of comorbid attention deficit hyperactivity disorder (ADHD) might have affected validity and reliability, as attention and reading comprehension issues were observed, especially in younger participants. Reduced attention spans may have led to inaccurate questionnaire responses, introducing potential bias. Moreover, some participants were receiving medication while others were not. All assessments were conducted under participants’ usual medication regimen and closely supervised by the researchers to ensure careful completion of the questionnaires. Nevertheless, certain medications may directly influence relevant processes such as empathy, emotion recognition, and emotional perception, representing a potential confounding factor (68). Third, unlike other studies, only self-report measures were used, increasing the risk of socially desirable responding. Fourth, the lack of a healthy control group limits the generalizability of the findings. Lastly, as the sample consisted solely of male participants, comparisons with previous studies, which often included mixed-gender samples, may be challenging. Future studies should make sure to include females to be able to study possible gender differences in the relationship between shame coping styles, social anxiety, and CU traits. Moreover, studies should also include community and outpatient samples to be able to extend the findings to more diverse samples.

## Conclusion and future directions

The present study provides evidence for the association between social anxiety and shame and shame coping in CD patients. In contrast, a direct association with CU traits was not detected, which might be due to developmental factors as well as sampling and measurement effects. In view of the inconsistent findings between the present study and the existing literature on the relationship between CU traits and shame (25), further research examining participants with an age range spanning from prepuberty to late adolescence is necessary in order to elucidate the influence of age. Future studies should explore CU traits and social anxiety while statistically controlling for CD, as has already been applied in previous research. Additionally, examining shame in a larger sample including community youth, outpatient settings and mixed-gender samples may clarify its relationship with CU traits. The inclusion of a healthy control group and an exploration of the high ADHD comorbidity in CD could further enhance our understanding of these associations.

## Data Availability

The raw data supporting the conclusions of this article will be made available by the authors, without undue reservation.
